# Periorbital Emphysema After Endoscopic Nasal Polyp Surgery

**DOI:** 10.4274/tjo.galenos.2018.01460

**Published:** 2019-02-28

**Authors:** Esat Çınar, Berna Yüce, Murat Fece, Fehmi Cem Küçükerdönmez

**Affiliations:** 1 Ekol Eye Hospital, Ophthalmology Clinic, İzmir, Turkey; 2 İzmir University of Health Sciences, Tepecik Training and Research Hospital, Ophthalmology Clinic, İzmir, Turkey

**Keywords:** Endoscopic surgeries, periorbital emphysema, valsalva maneuver

## Abstract

Periorbital and subcutaneous emphysema after transnasal endoscopic surgery are rare. Periorbital emphysema has been reported after facial trauma, dental interventions, procedures such as endoscopic sinus surgery and rhinoplasty, and due to medications such as systemic steroids. Although very rare, it may require urgent intervention because of the risk of increased intraocular pressure and impaired blood supply to the globe. The otolaryngology department requested ophthalmology consultation for a 65-year-old male patient who had severe periorbital emphysema of the right eye the day after endoscopic nasal polypectomy due to severe coughing and straining. Crepitus was detected on skin palpation and immediate intervention was performed by passing a 21-gauge needle through the skin into the subcutaneous tissue of the upper and lower eyelids to evacuate the subcutaneous air. The patient’s clinical symptoms resolved with no postoperative complications.

## Introduction

Periorbital emphysema is a clinical condition characterized by the accumulation of air beneath the skin around the orbit after surgery. It has been reported after maxillofacial surgeries, dental interventions, endoscopic sinus surgeries, and procedures such as rhinoplasty.^1,2,3,4^ Although periorbital emphysema is rare, it can lead to conditions that require urgent decompression. In this case report, a patient with periorbital emphysema was evaluated.

## Case Report

**Figure 1:**
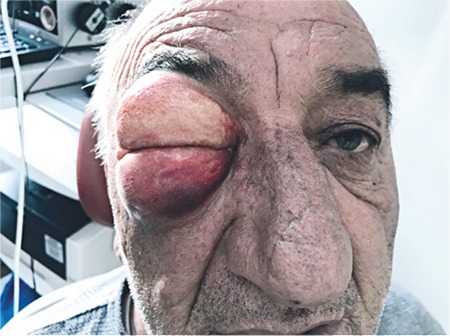
Periorbital emphysema involving the upper and lower eyelids of the right eye

**Figure 2:**
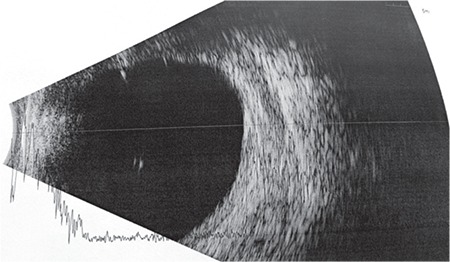
B-mode ultrasonography before surgical intervention

A 65-year-old man was referred by the otolaryngology department to our outpatient clinic due to sudden swelling and mild pain around the right eye. On examination, the patient exhibited what appeared to be severe edema encompassing the upper and lower lids of the right eye (Figure 1). Crepitus was clearly audible on palpation of the eyelids. An attempt to open the lids was unsuccessful. Visual acuity and intraocular pressure could not be measured due to extreme lid swelling. The patient reported that he had undergone transnasal endoscopic nasal polypectomy through the right nostril 2 days earlier. He said he had been instructed not to cough or strain after the endoscopic nasal surgery and the sudden swelling occurred immediately after severe coughing and straining. We suspected that the sinus wall was weakened due to his endoscopic surgery and the increased pressure caused by straining had forced air in the nose into the periorbital area. B-mode ultrasonography showed trapped air in the periorbital area (Figure 2). 

**Figure 3:**
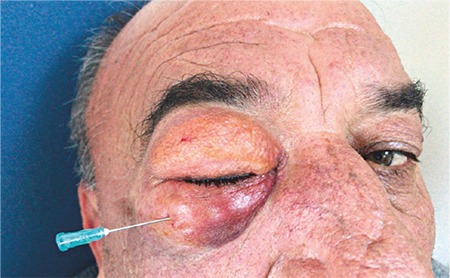
Marked reduction in lid swelling 5 minutes after surgical intervention

Considering the patient’s anxiety, the severity of periorbital emphysema, inability to conduct a full ophthalmologic examination, and the risk of complications such as compressive optic neuropathy, the patient was re-evaluated for a surgical intervention. After consultation, it was decided to evacuate the air using a 21 gauge needle inserted in the subcutaneous tissue of the upper and lower lids. In sterile conditions, the eye area was cleaned with 10% povidone-iodine. A 21-gauge needle was passed through the skin and subcutaneous tissue of the upper and lower lids parallel to the tarsus about 1.5 cm from the lid margin. Evacuation of subcutaneous air was evident from a significant reduction in lid swelling during the procedure (Figure 3). The patient’s vital signs were stable and the procedure was concluded. He was discharged with systemic antibiotics (cefuroxime axetil 500 mg twice daily) and moxifloxacin drops four times daily. 

**Figure 4:**
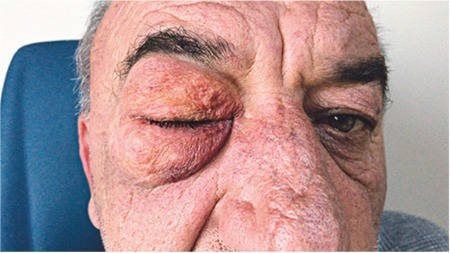
Appearance 1 day after surgical intervention

**Figure 5:**
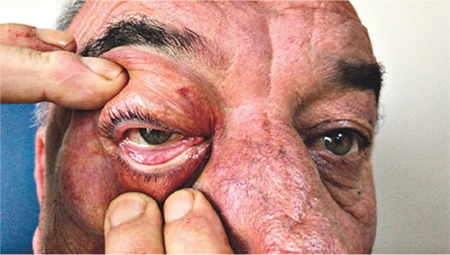
Appearance 1 day after surgical intervention

On follow-up examination the next day, the periorbital emphysema was substantially reduced and the globe could be examined (Figures 4, 5). He had full visual acuity in both eyes; intraocular pressure was 17 mmHg in the right eye and 16 mmHg in the left eye. Dilated fundus examination was normal. No restriction in eye movements was observed. Follow-up examinations at 1 week and 1 month revealed no pathological findings.

## Discussion

Trapped air in the periorbital subcutaneous tissue has been described in the literature using various terms such as subcutaneous emphysema, surgical emphysema, and interstitial emphysema.^5,6,7^ Although periorbital emphysema is usually associated with surgery, infection, or traumatic orbital wall fractures, spontaneous cases and other causes such as heavy lifting have also been reported. One of these reports described a 23-year-old male who developed periorbital emphysema in his left eye while lifting weights at a gym. No orbital wall fracture was detected on computed tomography and the periorbital emphysema regressed after 7 days with antibiotic and non-steroidal anti-inflammatory treatment.^8^

Periorbital emphysema is an uncommon clinical presentation that usually does not lead to major problems or require surgical decompression; however, in rare cases surgical decompression may be considered due to the potential for increased intraocular pressure and impaired ocular perfusion.^9^ In addition, it is important to differentiate periorbital emphysema from conditions like angioedema, anaphylactic reaction, and orbital cellulitis in order to avoid unnecessary antibiotic and antihistaminic treatment. In one case report, a patient developed swelling around the left eye after undergoing a dental procedure under local anesthesia. It was mistaken for an allergic reaction to the local anesthetic and the patient was discharged with antihistaminic therapy. However, she returned 6 hours later unable to open her eye and was found to have emphysema of the face and neck.^10^ Crepitus caused by air bubbles in the subcutaneous tissue is an important differential finding.^11^ Normal body and skin temperature and mild or absent pain are important in the differential diagnosis with orbital cellulitis. Our patient had pronounced crepitus and normal skin temperature. Imaging of the periorbital structures is also important for differential diagnosis. B-mode ultrasonography demonstrated trapped air in our case. Magnetic resonance imaging or computed tomography of the orbit can also be used to visualize emphysema.^12^

The use of systemic antibiotics in these patients is controversial. The general opinion is that systemic antibiotic prophylaxis is necessary in cases associated with oral or nasal surgery due to concern that staphylococci, streptococci, and anaerobic bacteria introduced via the same route as the emphysema (through a compromised bony wall) may cause periorbital infection.^13^ For this purpose we treated our patient with cefuroxime axetil, an oral cephalosporin with good soft tissue penetration.

A symptomatic approach is preferred for treatment. Painkillers can be given if there is pain, and local or systemic antibiotic therapy can be used if there are signs of infection. The condition tends to resolve within a week, but life-threatening conditions such as cardiopulmonary embolism, cardiac tamponade, and respiratory distress may occur depending on the amount of air and the deep fascial plane where it is trapped.^14^ Our patient displayed much more severe periorbital emphysema compared to images of other patients reported in the literature. This combined with his anxiety and diabetes led to our decision to perform urgent surgical intervention in order to reduce the risk of embolism. Although we believe that this decision was appropriate in this case to provide more rapid clinical improvement, eliminate pressure on the globe, and reduce the risk of embolism, we believe similar cases can be followed with a nonsurgical, symptomatic approach.

Periorbital emphysema has been reported after maxillofacial surgeries, dental interventions, and endoscopic sinus surgeries.^1,2,3,15^ Generally, air in the nasal cavity leaks into the periorbital space through a path in the deep orbital structures resulting from weakness or fracture in the bony structures due to surgical trauma.^16^ When questioned, our patient said he had also previously undergone endoscopic sinus surgery and nasal polyp surgery, both by transnasal approach. After these two previous endoscopic surgeries, the third operation may have caused weakness in the sinuses and bony structures of the nasal wall. We believe that when the patient increased the intranasal pressure with severe coughing and straining, air was forced through these weak tissues and into the periorbital area. In a study evaluating 137 cases of periorbital edema, it was reported that periorbital emphysema was more common in surgical procedures involving the orbital medial wall (78%).^17^ Because ultrasound clearly demonstrated the air trapped in the periorbital area in our patient, no additional imaging was performed. In a study of 1658 patients who had endoscopic sinus surgery, the incidence of ophthalmologic complications was 0.66%, with the most common being periorbital ecchymosis with or without periorbital emphysema (0.3%). The main risk factors to which the authors attributed complications were extension of primary disease, previous surgery, and anticoagulant therapy.^18^

Periorbital emphysema can also occur after ocular surgeries. Globe perforation, deep orbital tissue damage, and subsequent periorbital edema have been reported during retrobulbar anesthesia in particular.^19^ The possibility of globe perforation should not be overlooked in patients who have undergone intraocular surgery.

Although periorbital emphysema usually resolves within a few days with follow-up or decompression, a fatal case of emphysema starting in the periorbital area after routine gastrointestinal endoscopy and progressing to the face, neck, and chest with no ocular findings of compression was reported.^20^

Periorbital emphysema may be a rapidly progressive and life-threatening complication, or a benign and spontaneously resolving clinical entity. Definitive diagnosis should be established quickly and, after evaluating the potential risks, the patient can either be managed with observation and symptomatic treatment or with a simple surgical intervention, as in this case.
